# Impact of Radiation on Invasion and Migration of Glioma In Vitro and In Vivo

**DOI:** 10.3390/cancers16233900

**Published:** 2024-11-21

**Authors:** Marina Santiago Franco, Susanne Raulefs, Daniela Schilling, Stephanie E. Combs, Thomas E. Schmid

**Affiliations:** 1 School of Medicine and Health, Department of Radiation Oncology, TUM University Hospital, Technical University of Munich, 81675 Munich, Germany; susanne.raulefs@tum.de (S.R.); daniela.schilling@tum.de (D.S.); stephanie.combs@tum.de (S.E.C.); 2 Institute of Radiation Medicine, Helmholtz Munich, 85764 Neuherberg, Germany; 3 Deutsches Konsortium für Translationale Krebsforschung (DKTK), Partner Site Munich, 80336 Munich, Germany

**Keywords:** glioblastoma, migration, invasion, motility, microenvironment, irradiation, radiotherapy

## Abstract

Glioblastoma (GBM) constitutes the most common primary brain tumor and it remains incurable despite therapeutic advances. The high infiltration/invasion potential of GBM cells is considered to be one of the reasons for the inevitable recurrence of the disease. Radiotherapy (RT) is part of the standard care for patients with GBM, and its benefits on overall survival are extensively reported. However, numerous preclinical studies show that X-ray irradiation can enhance the motility of GBM cells. In the present review, we bring together state-of-the-art research on the impact of radiation on GBM cell motility. The mechanisms through which irradiation impacts the brain tumor microenvironment and the tumor cells themselves, leading to more aggressive/invasive tumors, are described. Finally, we summarize potential pharmacological strategies to overcome this problem. Clinical data validating the occurrence of these processes are urgently needed as they could be of great value for patient outcomes. With this comprehensive review, we expect to highlight the need for methods which allow for monitoring the post-irradiation invasive behavior of GBM in patients.

## Simple Summary

The most common primary brain tumor is glioblastoma (GBM), which remains incurable despite therapeutic advances, mainly because of its high infiltration/invasion potential. Numerous preclinical studies indicate that X-ray irradiation, part of the standard care for patients with GBM, can enhance the motility of the tumor cells. This review discusses the impact of irradiation on both the brain tumor microenvironment and GBM cells themselves and how it leads to enhanced invasion and migration.

## 1. Introduction

Glioblastoma (GBM) is the most common primary brain tumor, accounting for around 49% of the malignant intracranial neoplasms. It is also one of the most aggressive malignancies, with a poor prognosis and a median survival of less than 2 years [1,2]. The annual incidence of GBM ranges from 3 to 5 per 100,000 people, being higher in males. Incidence is also significantly affected by age, with cases increasing in adults over 40 years of age and peaking on those over 75 years [2].

A combination of surgery, radiotherapy (RT), and chemotherapy (temozolomide) is the current standard of care but, despite therapeutic advances, GBM remains incurable [3]. Treatment failure is attributed to the unique molecular characteristics of GBM. Among these are the population of glioma stem cells that make the tumor resistant to chemo- and RT and the high infiltration/invasion potential of GBM cells [3].

For patients with GBM, surgery and the extent of resection play the most important role in overall and progression free survival. However, a good functional outcome is necessary [4]. The complete surgical resection of the tumor is impossible without causing permanent neurologic morbidity because of the characteristic diffuse brain invasion of GBM. This diffuse infiltration also limits the radiation doses that can be delivered safely to the patients without permanent radiation damage of the surrounding healthy tissue [5]. Therefore, recurrence of the disease is considered inevitable, and the median progression free survival is around 7 months [1]. GBM cells usually invade as single cells, exhibiting a mesenchymal mode of invasion, by which cells generate protusions by readjusting their cytoskeleton and create strong adhesion forces with the extracellular matrix (ECM) by concentrating integrins on the cell surface [6]. The invasion mechanisms depend not only on intrinsic cell GBM cell characteristics but also on the communications between these cells and their microenvironment [7]. Migration and invasion using white-matter tracts and the perivascular space are considered the main features of GBM spreading, while extracranial metastases are extremely rare [3,6,8].

**Figure 1:**
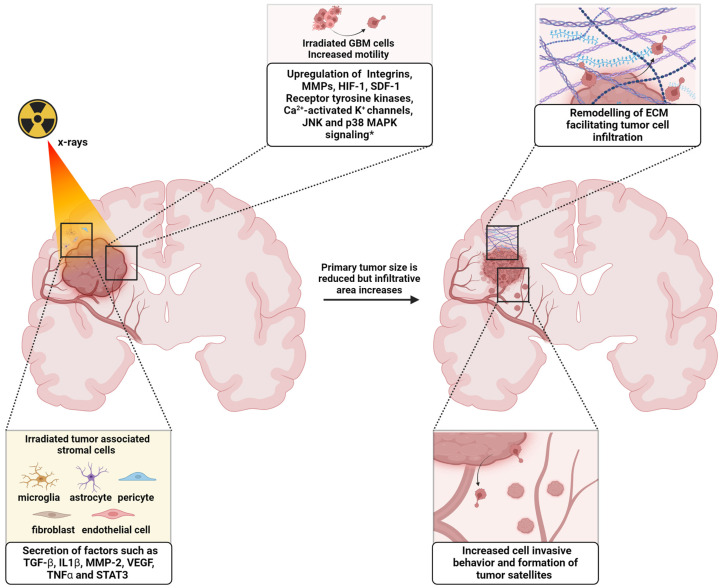
Radiation affects glioblastoma cells and their microenvironment. A reduction in the primary tumor size is often followed by increased infiltrative area due to enhanced tumor cell motility and remodeling of the extracellular matrix. * Upregulation of factors observed in vitro and/or in vivo. Created in BioRender. BioRender.com/q23e976 accessed on 14 November 2024.

RT is a keystone treatment for many tumors, including GBM, with its benefits on overall survival extensively reported. However, several studies point out that many different cancer cell types that are resistant to ionizing radiation might present enhanced migratory properties [9]. A better understanding of the impacts of prior RT to the brain on recurrent tumors is necessary. This might provide opportunities to target mechanisms that may promote the aggressiveness of GBM [10]. In the present review, we report findings on the impact of irradiation on GBM cells and their microenvironment and how it affects GBM migration and invasion abilities, as represented in Figure 1.

## 2. Radiation-Induced Changes in the GBM Microenvironment

An additional challenge to understanding response to RT in general and GBM in particular is the heterogeneity of GBM itself and the heterogeneity of the tumor microenvironment (TME) [11]. The heterogeneity exists not only between patients but also within a single tumor. The TME of GBM consists of cancer cells, different resident brain and immune cells (microglia), circulating immune cells (tumor-associated macrophages and tumor-infiltrating lymphocytes), and the brain vascular system. Tumor-associated macrophages are the predominant immune population in GBM and together with resident microglia can constitute about 30% of the tumor mass. The numerous cytokines released by these cells create the characteristic immunosuppressive TME of GBM, essential for its initiation and progression [12,13]. Understanding the post-RT responses by the immune system and identifying the source of inflammatory mediators and how they affect molecular pathways and cell dynamics is crucial to understanding tumor recurrence after treatment [14].

The TME also contains noncellular components such as signaling molecules, exosomes, components of the ECM, and its secreted remodeling enzymes. The diversity of components explains the dynamic and important role of the TME in cancer cell survival and resistance to therapy. Through direct cell-to-cell interaction or indirect release of cytokines and growth factors, cancer cells develop several strategies to hijack the TME in their favor. This reprograming of the TME by GBM cells facilitates their fast proliferation, migration, and invasion [15].

In a recent study, Watson et al. (2024) used a new technique (Hyperplexed Immunofluorescence Imaging) to analyze the modifications of the TME in response to irradiation [16]. They compared the GBM microenvironment response to that of a breast-to-brain metastasis using in vivo models. Both models presented similar survival benefits from the radiation treatment. However, an extensive reorganization of structural architecture, cell landscape, and spatial relationships in response to irradiation was observed only for GBM and not brain metastasis. This highlights the unique characteristic phenotype of GBM and how the irradiated environment can promote its dormancy and survival. Consequently, high rates of tumor relapse are observed, despite the initial RT benefit [16].

### 2.1. Radiation Impact to Cellular Components

Tumor-associated stromal cells (microglia, astrocytes, pericytes, fibroblasts, and endothelial cells) can also promote GBM progression. When a tumor is irradiated, these cells experience the therapy at the same levels as the tumor cells. For this reason, the stromal cell compartment must be considered when addressing RT-induced remodeling of the TME [17,18]. The review by Berg and Pietras (2022) provides an overview of the effects and consequences of radiation on different stromal cell types and reports how their response to RT promotes GBM cell migration, invasion, and proliferation through changes in the TME [18]. In the present review, we present data from in vivo studies that support the role of different stromal secreted molecules (e.g., TGF-β, IL1b, MMP-2, VEGF, TNFα, and STAT3) on GBM-enhanced cell motility post-irradiation.

### 2.2. Radiation Impact to Tumor Vasculature

Tumor vasculature communicates with the cellular components of the TME and the ECM contributing to tumor progression. GBM hypervascularization not only is involved in tumor cell nutrient supply and survival but is also closely related to its invasiveness and progression [19]. In the TME of GBM, cancer stem cells are directly involved in vessel formation by differentiating into endothelial cells or pericytes. Anti-angiogenic treatments targeting the VEGF/VEGFR signaling cascade such as bevacizumab are being investigated for GBM treatment. However, it has been demonstrated that GBM can re-activate angiogenesis even under the pressure of VEGF inhibition, suggesting the presence of compensatory angiogenic signaling pathways [20]. Vascular remodeling is an important hallmark of irradiation. Deshors et al. (2019) and Muthukrishnan et al. (2021) report how irradiation can lead to endothelial transdifferentiation of GBM stem-like cells, a treatment-induced tumor adaptation [21,22]. The study by Seo et al. (2019) showed radiation-induced changes in the tumor vasculature and microenvironment leading to therapy resistance [23]. VEGF also plays a role in GBM cell motility modulation and the impact of irradiation on VEGF secretion is further discussed in this review.

### 2.3. Radiation Impact to Noncellular Components

The ECM is the acellular component of the TME, comprising different glycoproteins, proteoglycans, and polysaccharides. It provides scaffolding for intercellular communication and cell migration, and additionally promotes tumor growth and cell invasion. The ECM should be considered as a non-static, active scaffold and an important player in the pathological remodeling of brain tissue after radiation therapy. Proteins involved in ECM biosynthesis, degradation, signal transduction, and ECM-glioma cell interactions have been reported to be upregulated upon radiation, as reviewed by Gupta and Burns (2018) [10]. This facilitates tumor cell infiltration which can lead to tumor recurrence after radiation therapy. RT-induced changes in the ECM are therefore potential therapeutic targets [10].

The hypoxic GBM environment is considered a key trigger for angiogenesis, survival, invasion, and resistance to therapies [15]. Radioresistance under hypoxic conditions is well characterized as DNA damage is diminished under insufficient amounts of reactive oxygen species (ROS) and/or reactive nitrogen species (RNS), responsible for 60–70% of DNA lesions [24]. The review by Monteiro and coworkers (2017) identified molecular mechanisms by which hypoxia-responsive proteins contribute to GBM migration and invasion [25]. Some of these proteins, such as those involved in ECM degradation (e.g., integrins, MMP-2, and MMP-9), were found to be upregulated in irradiated GBM cell lines and in vivo GBM models, as will be further discussed herein.

## 3. The Impact of Radiation on GBM Cell Motility In Vitro

### 3.1. In Vitro Models

In experimental cell biology, migration and invasion are separated terms. Migration is the non-destructive, non-proteolytic movement of cells in surfaces where no obstructive fiber network is present. On the other hand, invasion is the movement through a three-dimensional (3D) matrix where the cell must not only modify its shape but also remodel the matrix [26]. Different in vitro models have been used to support the study of GBM cell motility, avoiding complex, expensive, and time-consuming experiments in animal models [27].

The models that are simplest to use consist in two-dimensional assays such as wound healing with silicone inserts and scratch assays using a pipette tip. In these models, a gap is created on a confluent cell monolayer and cells are allowed to migrate horizontally. They are simple and useful to evaluate the migratory abilities of cell masses, although the cells are separated from their natural microenvironment [28,29].

Three-dimensional models can better mimic tumor development and heterogeneous areas. Transwell inserts containing porous membranes can be used to evaluate cell migration and/or invasion and give important information on the abilities of single cells to respond to treatments. For migration assays, the membrane is not coated but it is important to highlight that not all cells that migrate horizontally are able to cross a pore membrane. For invasion assays, the membrane is coated with Matrigel or other extracellular matrix components, which block non-invasive cells from migrating through. On both assays, cells are seeded and allowed to migrate/invade to the other side, which might contain or not a chemoattractant [28,29]. During spheroid migration assay, cells move concentrically outward of a cell spheroid placed on a tissue culture dish. The spreading area can be measured over time. This assay mimics tumor biology with close cell-to-cell contacts and different supplies of nutrients and oxygen to cells in different spheroid locations. Spheroids can also be embedded in a matrix to allow for spheroid invasion assays [26].

The different studies performed so far to investigate GBM cell migration and invasion in vitro are presented in Table 1 and Table 2, respectively. For evaluating cell migration, different studies have been reported such as scratch assay, transwell migration, time-lapse videography, and spheroid migration. All invasion studies were Matrigel-coated transwell invasion assays with an 8.0 μm porosity, with the exception of the study by Eke and coworkers (2012), which consisted of spheroids embedded in a collagen type I matrix [30] and the study of Nakamura and coworkers (2007) using a co-culture assay, which allowed for the quantitation of human glioma invasion into a background of normal human astrocytes [31]. Most of these studies point towards an increased migratory/invasive capacity triggered by X-ray irradiation.

### 3.2. Molecular Mechanisms

Besides the measurements of cell motility, molecular pathways were investigated in many of these studies.

#### 3.2.1. MET Oncogene

Bacco and coworkers (2011) observed increased expression of the mesenchymal–epithelial transition factor (MET) oncogene in GBM cells after irradiation [32]. MET is known to play a critical role in enhancing migration, invasion, therapeutic resistance, and recurrence of glioblastomas [59].

#### 3.2.2. Notch Signaling Pathway

Kumar et al. (2022) demonstrated a radiation-induced upregulation of Notch signaling, a pathway that is heavily involved in the progression of gliomas [33]. Its dysregulation promotes epithelial–mesenchymal transition (EMT) and contributes to maintaining glioma cell stemness, enhancing migration and invasiveness [60,61]. This happens most likely through Notch1-mediated downregulation of epithelial markers such as E-cadherin and upregulation of mesenchymal markers such as Snail and Vimentin [62]. Enhanced expression of vimentin in GBM cell lines after irradiation was reported by Kouam et al. (2018) [47].

#### 3.2.3. Cathepsins

Secreted cathepsins can degrade the extracellular matrix (ECM), altering the TME, and also disrupt cell–cell adhesion molecules contributing to invasion and metastasis [63]. Xiong and coworkers (2017) demonstrated that enhanced cathepsin L expression is involved in the radiation-induced migration and invasion of GBM cells [35].

#### 3.2.4. DNA-PKcs

DNA-dependent protein kinase catalytic subunit (DNA-PKcs) has been primarily investigated as a prominent component of non-homologous end joining (NHEJ) repair. Recent research suggests the promotion of cell migration and invasion as one of the non-canonical roles of DNA-PKcs contributing to tumorigenesis [64]. Liu et al. (2021) demonstrated that irradiation enhanced the migration and invasion of DNA-PKcs-positive (M059K) but not DNA-PKcs-negative (M059J) cells [36].

#### 3.2.5. Integrins

The role played by integrins on adhesive, migratory/invasive properties of glioblastoma has been extensively studied [65]. Among them, integrin-αvβ3 was the first to be reported as abundantly expressed in GBM, with nearly 60% of tumor samples expressing it, whereas the normal brain tissue does not. Integrin-αvβ3 is also preferentially expressed at the invasion front of the tumors, highlighting its important role in invasiveness [66]. Different in vitro studies showed that irradiation increased the expression of integrins leading to an increase in migratory/invasive potential [40,44,48]. Wild-Bode and coworkers (2001) showed that although integrin-αvβ3 is involved in irradiation-enhanced cell migration, its role is not specific for irradiation-induced motility [48]. This has also been shown for β1 integrins on a study by Eke and coworkers (2012) [30].

#### 3.2.6. MMPs

It has been shown that glioma tissue is one of the main sources of matrix metalloproteinase (mostly MMP-2 and MMP-9) as compared to normal brain tissue [66]. These proteolytic enzymes are involved in tissue remodeling by degradation of numerous ECM proteins [67].

The expression of integrin-αvβ3 was found to colocalize with (MMP-2), interacting and modulating its activity [65,66,68]. Cordes et al. (2003) demonstrated that irradiation increased β1- and β3-integrin expression as well as MMP-2 expression on glioma cells. These integrins were shown to play an important role in invasion and MMP-2 activity [58].

Badiga et al. (2011) showed that irradiation enhanced MMP-2 expression on glioma cell lines [49]. Increased expression of both MMP-2 and MMP-9 post-irradiation was observed by D’Alessandro and coworkers (2019) [42] and by Dong and coworkers (2015) [59]. Irradiation induced MMP-2 activity in conditioned cell culture media of a GBM cell line was reported by Furmanova-Hollenstein et al. (2013) [54].

Park and coworkers (2006) demonstrated that the effects of irradiation on MMP-2 secretion might be dependent on PTEN status. In cells lacking functional PTEN, irradiation enhanced MMP-2 secretion, which was not observed for cells harboring WT-PTEN. This impacted the post-irradiation invasion outcome, which was increased only in cells lacking functional PTEN [56].

#### 3.2.7. Receptor Tyrosine Kinases

The in vitro studies presented herein also demonstrate the role of different members of the receptor tyrosine kinases (RTK) family, such as the vascular endothelial growth factor receptor (VEGFR); the insulin-like growth factor receptor-1 (IGFR-1); and the epidermal growth factor receptor (EGFR), in irradiation-induced GBM cell motility.

Studies with glioma cell lines report that irradiation can stimulate the VEGF secretion in a cell line-dependent manner. In a study by Kil et al. (2012), it was demonstrated that conditioned medium from irradiated cells led to an enhancement in cell motility due to radiation-induced VEGF secretion [41]. Krcek and coworkers (2017) figured out that an increase in VEGF secretion after irradiation can lead to a synergistic effect between VEGF and irradiation on migration velocity [46]. Enhanced expression of VEGF post-irradiation was also observed by Dong and coworkers (2015) [55].

IGFR-1 stimulation by IGF-I promotes glioma cell growth, proliferation, and migration, triggering the progression of low-grade glioma to GBM [69].

EGFR is the most amplified of any gene in GBM and is known to be mutationally active in over 50% of tumors, contributing to cell motility and disease progression [70,71]. D’Alessandro and coworkers (2019) observed enhanced EGFR gene expression after irradiation [42].

#### 3.2.8. Calcium-Activated Potassium Channels

An altered potassium flux is essential for GBM cell motility. During cell migration, fluctuations in the cytosolic calcium concentration are observed demanding calcium-activated potassium channels such as KCa3.1 (intermediate conductance K+ channel) and the BK channel (large conductance K+ channel). These are overexpressed in 32% of glioma patients, and a linear correlation between their expression and the progression of the disease is observed [72]. Steinle et al. (2011) reported BK channel activation after irradiation of glioma cell lines playing an important role in radiation-induced migration [39].

#### 3.2.9. miRNAs

A number of microRNAs (miRNAs) are dysregulated in GBM. One notable miRNA consists of miR-10b, which is strongly upregulated in gliomas but absent in normal human brain tissue. MiR-10b can promote the migration and invasiveness of different tumor cells including glioma [73,74]. Against the majority of studies reporting enhanced motility after exposure to radiation, Zhen and coworkers (2016) observed a reduction on both migratory and invasive behavior following irradiation of cell lines with 5 Gy. In order to evaluate the role played by miR-10b, cells transfected with a miR-10b mimic were irradiated with the same dose and migration/invasion was quantified. They verified that miR-10b enhances motility of GBM cells when irradiated as compared to non-transfected irradiated cells [45].

#### 3.2.10. JNK and p38 MAPK Signaling

Jun N-terminal kinase (JNK) and p38 mitogen-activated protein kinase (p38 MAPK) signaling plays a role in cancer progression and metastasis. Enhanced p38 activity has been detected in GBM cell lines and human glioma samples, while JNK phosphorylation correlates with glioma histological grade [75].

#### 3.2.11. MDA-9/Syntenin

Melanoma differentiation associated gene–9 (MDA-9/syntenin) has roles in cell–cell and cell–matrix adhesion and has been shown to act as an important mediator of GBM invasion through the activation of NF-kB via a c-Src-dependent pathway [76]. MDA-9/syntenin expression in human-derived GBM cell lines and patient samples increases with tumor grade and correlates with lower response to RT and poorer prognosis [52].

## 4. Radiation-Induced GBM Invasion In Vivo

Rodent brain tumor models are valuable tools for investigating therapies for GBM. Regarding rat brain tumor models, the C6 glioma, 9L gliosarcoma, and F98 glioma models are the three most used in GBM research. Mouse brain tumor models are numerous, ranging from the most frequently used glioma cell line GL261 in immunocompetent mice to human glioma xenograft models, mostly using the U87 glioma. Although these models do not fully recapitulate human GBM for different reasons, they have already aided in the development of human GBM treatments [77].

Different in vivo studies were performed with the aim of assessing the contribution of brain irradiation to the invasive profile of glioma cells, as listed in Table 3.

Wild-Bode and coworkers (2001) injected pre-irradiated (1 or 3 Gy) or non-irradiated 9L cells orthotopically in rats that were sacrificed 21 days post-injection and had their brains collected. Tumor volumes were determined in histology sections stained with H&E and no significant difference was observed between the two groups. Brain sections were stained with nestin antibody (a cellular marker for infiltration) for determination of invasiveness and nestin-positive cell clusters (>10 cells) distant from the primary tumor mass were counted. This revealed that tumors formed from pre-irradiated 9L cells were more invasive and presented more distant clusters than the ones formed from non-irradiated cells. A higher expression of MMP-2 (matrix metalloproteinase) and lower expression of TIMP-2 (tissue inhibitor of metalloproteinases) was also observed in tumors originated from pre-irradiated cells [48].

In a similar study, Zhai et al. (2006) investigated the impact of radiation treatment on cell invasion in a rat model injected orthotopically with either pre-irradiated (3 Gy) or non-irradiated 9L glioma cells [43]. At 21 days post-implantation, the degree of tumor invasion was assessed histologically in brain sections stained with nestin antibody. The invasion index was calculated based on the number of tumor cell clusters occurring far from the primary tumor site. It was observed that irradiation increased the invasion index two-fold as compared to the control. They also evaluated the effects of pre-treatment of the cells with inhibitors (25 μM) of IGFR-1, PI-3K, and Rho. These pre-treatments significantly reduced (five-to-six-fold) the irradiation mediated invasion, indicating they might be important targets to improve the outcomes after radiation treatment [43].

Tabatabai and coworkers (2006) were the first to evaluate whether irradiation would induce the invasiveness of preformed gliomas in an animal model [78]. Using an LNT-229 orthotopic xenograft mouse model, they observed that irradiation of the tumors with 8 Gy led to the formation of cell satellites distant from the bulk tumor. Upregulation of MMP-9 and HIF-1α was observed in sections of the irradiated tumor as compared to the control [78].

Desmarais et al. (2012) investigated the contribution of brain irradiation to the infiltration profile of glioma cells [79]. An F98/Fischer rat glioma model that is known to properly reproduce the characteristics of human GBM was used. In this study, they irradiated either the brain, the cells, or both, aiming to separately investigate the effects of irradiation. Groups consisted of (1) animals that were injected with non-irradiated F98 cells (control); (2) animals that were injected with pre-irradiated F98 cells (FR IR); (3) animals which had the brain pre-irradiated before injection of non-irradiated F98 cells (Brain IR); (4) animals that had the brain pre-irradiated before injection of pre-irradiated F98 cells (Brain IR + F98 IR). Brain irradiation consisted of a whole-brain single dose of 15Gy delivered 14 h before tumor implantation. Cell irradiations consisted of irradiating the cells with the LD50 one hour before orthotopic tumor injection. It was concluded that the pre-irradiation of F98 cells before injection did not affect their migratory capacity in the brain parenchyma. However, pre-irradiation of the brain before injection of either non-irradiated or pre-irradiated F98 cells led to a switch from a proliferation to an infiltration phenotype. This was shown by a reduction in the primary tumor growth but an increase in the infiltrated surface [79]. Nestin was evaluated and its expression was increased in the infiltrative cells of tumors growing in pre-irradiated brains. They also observed increased levels of TGF-β1 and IL-1β after brain irradiations, cytokines that are involved in pathways that lead to enhanced migration/invasion. It was then proposed that increased levels of these cytokines were responsible for the elevated levels of PGE2 and PGD2 as well as COX-2 observed in irradiated brains. PGE2 is known to stimulate the production of MMP-2, and these higher levels of the latter were also observed in this study. MMP degrades extracellular matrix proteins, explaining the higher infiltrative profile of the glioma cells on the pre-irradiated brains. It was concluded that brain irradiation before tumor cell injection leads to alterations in tumor growth, which culminate in increased aggressiveness and higher lethality. The median survival for animals in control and F98 IR groups were 25.3 and 24 days, respectively. However, the survival for Brain IR and Brain IR + F98 IR groups was significantly shorter: 18.2 and 21.3 days, respectively [79].

Wang and coworkers (2013) implanted ALTS1C1 glioma cells orthotopically in mice and 13 days later, tumors were irradiated with a single dose of 8 Gy or 15 Gy [80]. The survival was determined as 24 ± 2 days for non-irradiated control mice and 28 ± 2 days and 30 ± 1 days for mice irradiated with 8 Gy and 15 Gy, respectively. Irradiation significantly reduced tumor size for mice irradiated with both 8 Gy (68% of control) and 15 Gy (64% of control). On the other hand, a significantly higher number of infiltrating satellites was present for irradiated groups compared to the control. They also observed that irradiation led to satellites with a higher microvascular density, higher number of infiltrating macrophages, and increased expression of stromal cell-derived factor-1 (SDF-1) and hypoxia-inducible factor-1 (HIF-1) as compared to the control [80].

Shankar et al. (2014) investigated the effects of irradiation on the proliferation, invasion, and migration of primary GBM cells obtained from an explanted tumor of a patient on nude rats with orthotopic tumors [81]. On day 84 post-tumor implantation, rats were irradiated with a single dose of 50 Gy and kept until day 133. The control group consisted of rats with non-irradiated tumors that were kept until day 112. Tumors from rats that received irradiation presented a higher proportion of Ki-67 positive cells, with a proliferation index around 2.5-fold higher than the control group. Denser and significantly larger areas of MMP-2 staining were also observed in tumors from irradiated rats in comparison to the control group, indicating greater invasive capacity. The cell surface molecule CD-44 is responsible for cell-to-cell and cell–matrix interactions. In this study, CD-44 positive cells were tracked for cell migration assessment. Significantly higher infiltration (>1.5-fold) was observed for the irradiated group compared to the non-irradiated [81].

Pei and coworkers (2015) investigated the effects of radiation on invasion of bioluminescent U87 cells injected orthotopically in nude mice [82]. Irradiation of tumors was performed 2 weeks after tumor implantation with a single dose of 6 Gy or five fractions of 2 Gy. Animals were killed on day 30. Histological examination of the brain tissues showed that exposure to irradiation increased tumor invasiveness and the number of tumor cell satellites. In situ zymography enables localization of gelatinolytic activities in histological sections and a signal was present around the margins of tumors from animals that were irradiated but not in the non-irradiated ones. Immunohistochemistry for MMP-2 revealed no difference between control and irradiated groups [82].

**Figure 2:**
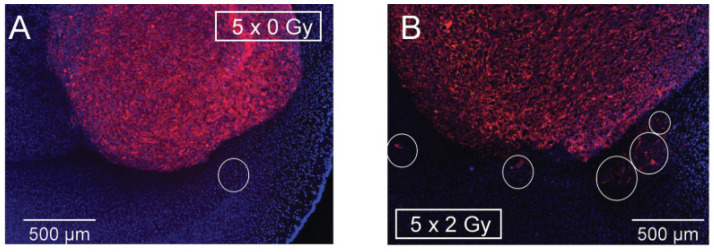
Effects of brain irradiation on U-87MG-Katushka orthotopic tumor development. Slices of non-irradiated brain (**A**) or irradiated ((**B**); 5 × 2 Gy) three weeks post-tumor treatment. Margins of non-irradiated tumors are clearly delimited while irradiated tumors present fringed margins with invasion zones. Circles highlight invaded cells. Reproduced from Edalat et al., 2016 [83].

Edalat and coworkers (2016) evaluated the radiation-induced infiltration of glioblastoma cells using an orthotopic immunocompromised mouse model of human glioblastoma (U-87MG-Katushka cells) [83]. Seven days post-tumor implantation, irradiation of right brain hemispheres was started in daily fractions of 2 Gy for a total of five fractions. Mice had their brains collected three weeks post-tumor challenge. Brain sections were evaluated for cell migration. Emigration activity was determined by counting the number of evaded cells as well as the migration distances. The total number of emigrated cells as well as the migrated distances were significantly higher on irradiated tumors, as shown in Figure 2 [83]. Glioblastoma cells are known to express high levels of Ca^2+^-activated BK K+ channels. These are suggested to play an important role in glioblastoma proliferation and migration [39]. The group further observed that the irradiation-induced cell migration was blocked when animals were treated systemically with the BK channel inhibitor paxilline (8 mg/Kg, 6 h prior and 6 h after each irradiation fraction). This suggests that BK channel activation was responsible for irradiation-induced glioblastoma invasion. A marked upregulation of stromal cell-derived factor-1 (SDF-1) in brain sections was also observed by immunostaining after fractionated irradiation. This suggests that SDF-1 can stimulate Ca^2+^ transients that lead to the observed BK channel activation [83].

Kegelman and coworkers (2017) investigated the impact of irradiation on invasiveness of orthotopic U1242-luc cell tumors in mice [52]. Treatment with 2.5 Gy irradiation for 4 consecutive days was started 11 days post-tumor implantation. The average survival for control mice was 41.3 days, while that for irradiated animals was extended to 62.8 days. Histological analysis revealed infiltrating tumors for both control and irradiated groups [52]. Melanoma differentiation-associated gene 9 (MDA-9/Syntenin) is involved in invasion and metastatic signaling in different tumors [88]. To identify its possible role as a regulator of GBM invasion, they administered PDZ1i (113B7), a specific inhibitor of MDA-9/Syntenin activity, 2 h before radiation treatment on each of the 4 treatment days. For animals treated with the combination of PDZ1i irradiation, survival was extended to 78.8 days and tumors presented margins that were markedly more delimited and less invasive. These results support the role of MDA-9/Syntenin as a mediator of post-irradiation invasion enhancement [52].

Birch and coworkers (2018) evaluated radiation-induced invasion in nude mice injected orthotopically with G7 patient-derived GBM cell line [84]. Tumors were allowed to grow and then irradiated with three fractions of 2 Gy. The quantification of GBM cells away from the primary tumor mass was performed in brain sections collected 17 days after starting the irradiation treatment. The results supported the concept that radiation promoted infiltration of GBM. They further investigated the potential role of myotonic dystrophy kinase-related CDC42-binding kinase (MRCK) on this radiation-induced migration. Therefore, they administered a small-molecule inhibitor (BDP-9066) of MRCK to the animals and observed that treated mice had no increase in tumor cell infiltration when irradiated as compared to the controls. This was translated into increased survival for the treated animals in an additional extended efficacy experiment. It was concluded that irradiation alone provided a survival benefit compared to the control, but this was significantly enhanced when combined with the MRCK inhibitor [84].

Zhang and coworkers (2018) used an orthotopic mouse glioma model with U251 cells and observed more invasive borders for tumors irradiated with three fractions of 5 Gy, and more satellite tumors for this group as compared to the non-irradiated control. STAT3 is a transcription factor known to contribute to different biological processes including migration and invasion. Histochemical analysis showed increased expression of phospho-STAT3 (Ser727) and phospho-STAT3 (Tyr705) as compared to the non-irradiated control [85].

**Figure 3:**
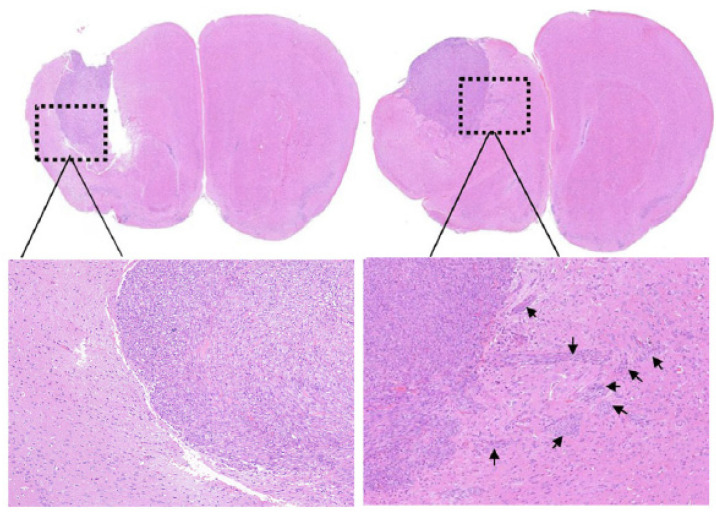
H&E staining images of mouse brain with orthotopic tumors four weeks after injection of control non-irradiated U87 cells (**left**) or pre-irradiated U87 cells (**right**). Black arrows indicate the disseminated tumors. Reproduced from Liu et al., 2020 [36] with permission from Elsevier.

Liu et al. (2020) injected pre-irradiated (2.17 Gy), pre-irradiated genistein-treated (2.17 Gy) or non-irradiated non-treated U87 cells orthotopically in mice that were sacrificed four weeks post-injection and had their brains collected [36]. Tumor histology sections were stained with H&E and for immunohistochemistry, tissue sections were stained with DNA-PKcs, Rac1, AKT2, MMP2, E-cadherin, and vimentin antibodies. H&E results (Figure 3) revealed irradiation-induced tumor invasion (present in 80% of mice), which was significantly reduced (20% of mice) when cells were pre-treated with genistein. Radiation induced enhanced expression of DNA-PKcs, AKT2, and Rac1, which was also suppressed by pre-treatment with genistein, indicating it suppresses the DNA-PKcs/AKT2/Rac1 pathway in vivo. Additionally, genistein reduced the expression of MMP2 and vimentin and enhanced the expression of E-cadherin induced by irradiation [36].

Tsuji and coworkers (2021) designed a study aiming to investigate the impact of irradiation on the microenvironment of brain tissue and its relation to the recurrence and progression of glioma [87]. Fisher rats had their right brain hemisphere irradiated with 65 Gy and after 3 months F98 glioma cells were injected orthotopically on the irradiated ipsilateral brain. The tumors themselves did not receive irradiation treatment. The median survival of animals growing tumors in the irradiated brain (20.5 days) was significantly lower when compared to that of animals growing tumors in non-irradiated brains (22.5 days). Irradiated rats without tumors were living for more than a year, showing that irradiation alone was not the cause of death of animals. Tumors growing on the irradiated brain showed a significantly higher cell proliferation index (Ki-67 antibody labeling), as well as tumor invasiveness (histological cuts), in comparison with tumors in non-irradiated brains. They also demonstrated that irradiation led to significantly higher expression of CXCL12, VEGF-A, TGF-β1, and TNFα in brain tissue in comparison to non-irradiated brains. Regarding the implanted tumor cells, higher expression of CXCR4, FGF-2, and ERK2 was observed in cells from tumors growing in previously irradiated brains in comparison to tumors implanted in non-irradiated brains. These results suggested that the microenvironment of irradiated brains promotes tumor cell replication at several months post-irradiation [87].

Stransky and coworkers (2023) evaluated the development of satellite tumors in a mice model with orthotopic SMA-560 cell tumors [37]. Seven days after tumor implantation, tumors were irradiated with 4 Gy for five consecutive days or animals were placed on control group. The median survival for tumor-bearing mice in the control group (16 days) was lower than that observed for irradiated mice (26 days) but not statistically significant. Up to 14 days after the end of treatment, tumor growth morphology was evaluated. It was observed that satellite tumors were more frequently found in the irradiated group (four out of five animals) than in the control group (one out of six animals), indicating the irradiation-induced invasiveness of SMA-560 cells. On in vitro studies performed with the SMA-560 cells (wound healing and transwell migration assays) irradiated with sham or 2 Gy, no differences were observed between these two groups. This allowed the authors to conclude that the in vivo findings were not due to an increase in intrinsic cell motility [37].

## 5. Limitations of the In Vitro and In Vivo Studies

It is important to keep in mind that cells cultured in plastic do not recapitulate physiologically important components and the dimensionality of human brain tissue. Therefore, in vitro models that better mimic the structure and functionality of this tissue are highly desired [89]. For example, no studies have been performed yet to investigate the impact of irradiation on glioma invasion using human brain organoids. These models have higher physiological relevance compared to 2D cultures or spheroids, as they recapitulate tumor heterogeneity. They allow the observation of the interactions of tumor cells with normal brain cells and could provide valuable information [90,91]. Some of the in vitro migration studies presented herein consisted of scratch assays. These are a simple, inexpensive protocol. However, as a scratch assay consists of a manually created wound, it leads to highly irregular scratches, which might contain an accumulation of removed cells on the edges. Additionally, if not performed carefully, it can impair the extracellular matrix coatings on the cell culture dish. Taken together, these limitations can have a great impact on the accuracy of the data collected via scratch assays [92].

Another limitation of in vitro studies is the fact that it is not always possible to distinguish between migration/invasion and proliferation when it comes to contributions to the study outcomes (gap closure, number of cells that cross transwell membranes or spheroid volumes) [93]. In some of the studies reported here, serum starvation was performed to avoid cell proliferation within the timeframe of the study, but in others, no such measure was taken. Moreover, it is important to use treatment doses which have no cytotoxic effect throughout the timeframe of the study, make it possible to distinguish between migration/invasion inhibition and cell death [56]. The studies of Zhai and coworkers (2006) and Goetze and coworkers (2007) reveal an increase in cell migration with lower doses and a decrease with higher doses (>8 Gy), which could be due to lower cell viability, which was not assessed [43,44].

Regarding animal models, they do not fully recapitulate the intratumoral heterogeneity of human gliomas, especially when immune-deficient animals are used with xenograft tumors where the selection for the faster growing cell clones reduces the intratumoral heterogeneity even further [94].

Additionally, some of the in vivo data herein reported were obtained with either cells or brains that received pre-irradiation before transplantation. In some, either whole-brain irradiation or large irradiation fields, which do not recapitulate what is possible in clinical scenarios, were delivered. In others, irradiation was delivered as a large single dose, which also does not properly reflect the clinical setting. Despite the different limitations, taken together the pieces of evidence collected strongly suggest that irradiation-induced migration/invasion is a general phenomenon and can play a role in the recurrence of GBM in patients [83].

## 6. Effect of Other Types of Ionizing Radiation on Migration and Invasion of GBM

One innovative radiotherapy treatment concept for patients with glioblastoma (GBM) is the use of carbon ions, which possess distinctive physical advantages, including the Bragg peak, which enables enhanced dose deposition to the tumor. As demonstrated in the study conducted by Rieken et al. (2011), carbon ions did not induce radiation-induced invasion or migration of GBM cells. In this study, it was observed that in comparison to photon RT, carbon ions led to a reduction in integrin expression and inhibition of glioma cell migration in vitronectin and fibronectin substrates [40]. This indicates that carbon ion radiotherapy may provide enhanced local control. Kumar and coworkers (2022) reported that, in contrast to photon RT, carbon ion irradiation downregulates Notch signaling, reducing the migration and spheroid formation of glioma cells [33]. Vashishta et al. (2023) reported a decrease in the migration of U251 cells after exposure to carbon ions as opposed to the dose-dependent increase in migration following photon RT [34]. A study by Wank et al. (2018) employed primary patient-derived cell lines to compare the effects of photon RT to those of high LET alpha particles on invasion. All 16 primary cell lines tested demonstrated a lack of radiation-enhanced invasion following high LET irradiation, in contrast to low LET irradiation [53]. Zaboronok et al. (2014) observed a similar tendency of proton beam irradiation on stimulation of migration and invasion of the glioma cell line U87MG. However, just this one cell line was evaluated in this study [95]. Nevertheless, to the best of our knowledge, only in vitro studies evaluating the impact of other types of ionizing radiation on GBM migration and invasion have been performed so far.

## 7. Pharmacological Approaches Targeting Basal and Radiation-Induced Migration/Invasion

### 7.1. Basal Migration/Invasion

Many therapeutic agents that target the molecular mechanisms contributing to migration/invasion of glioblastoma have been developed [96,97]. Preclinical in vitro and in vivo data investigating inhibitors, e.g., against MMPs, ADAM10, integrins, Notch signaling, TGF-ß, PI3K, gap junctions, ion channels, and other molecular targets, have revealed promising anti-invasive activity. However, so far, clinical trials aiming to prevent the invasion of GBM by inhibition of, e.g., integrins (cilengitide), TGF-ß (galunisertib), or Notch (RO4929097), have failed to show prolonged overall survival (OS) or progression-free survival (PFS) [98,99,100].

Interestingly, the YAP-TEAD inhibitor verteporfin, an FDA-approved drug for macular degeneration, has demonstrated an impressive anti-invasive effect and a survival benefit in patient-derived orthotopic xenograft GBM models [101]. Therefore, a phase 1/2 clinical study with liposomal verteporfin in recurrent glioblastoma has been initiated to repurpose this drug (NCT04590664) [102].

Of course, further clinical trials with novel molecular-targeted drugs against glioma invasion are currently underway [97].

### 7.2. Radiation-Induced Migration/Invasion

Only a few in vitro and in vivo studies have addressed the inhibition of radiation-induced invasion or migration.

The natural isoflavone genistein abrogated radiation-induced invasion and migration of GBM cells in vitro and in vivo by binding to DNA-PKcs and blocking the DNA-PKcs/Akt2/Rac1 pathway [36].

Eke and coworkers (2012) showed that anti-integrin b1 antibodies (clone AIIB2) diminished the migration of certain glioblastoma cell lines independently of irradiation. Inhibitors of JNK (SP600125) and p38 MAPK (SB-203580) significantly impaired GBM cell invasion as compared to controls [30]. Constitutive and radiation-induced migration and invasion of GBM cells has been abolished by the integrin inhibitor echistatin [103].

Treatment with inhibitors against Src, EGFR, p38, PI3K, and Akt counteracted the radiation-induced MMP-2 upregulation and subsequently the invasion of mutant PTEN glioma cells in vitro [56]. Furmanova-Hollenstein et al. demonstrated that patupilone, a microtubule stabilizer, reduces the radiation-induced MMP activity and invasion of U251 GBM cells in vitro [54]. XAV 939, which leads to degradation of ß-catenin, decreased radiation-induced MMP expression and thereby abrogated the pro-invasive effect of radiation in U87 GBM cells [55]. Badiga et al. (2011) showed that inhibition of MMP-2 by transfection using plasmid constructs carrying siRNA against MMP-2 (p-MMP-2) led to reduced migration and invasion of glioma cell lines [49].

The inhibition of the IGFR-1 signaling pathway as well as the inhibition of EGFR led to a significant reduction in irradiation induced cell motility [43].

D’Alessandro and coworkers (2019) reported that the inhibition of the potassium channel KCa3.1 by TRAM-34 reduced the irradiation-induced expression of many invasion-related genes (CXCL12, CXCR4, MMP2, MMP9, MMP12, EGFR, KCNN4, AP-1, ATF2, EGR3, REST) abolishing the radiation-induced pro-invasive phenotype [42]. As observed by Stransky et al. (2023), TRAM-34 blocked the radiation-induced hyper-invasion of glioma in an orthotopic mouse model and in combination with irradiation prolonged the survival of mice [37].

Pharmacological targeting of melanoma differentiation-associated gene 9 (MDA-9/Syntenin) by PDZ1i inhibited radiation-induced invasion and radiosensitized GBM in vitro and in vivo [52]. PDZ1i downregulated Src and EGFRvIII signaling and reduced the secretion of MMPs following radiation.

Very interestingly, gadolinium chelate-coated gold nanoparticles revealed anti-invasive effects on irradiated GBM cells in vitro and in vivo and exerted radiosensitization [104].

YM155 decreased radiation-induced invasion in U251 and U87 GBM cell lines in vitro through inhibition of STAT3 [85].

Invadopodia formation is known to contribute to the invasive phenotype of cancer cells [105]. Irradiation and also temozolomide treatment can increase invadopodia activity in GBM [106]. A screening of FDA-approved drugs for their “anti-invadopodia” effects showed that paclitaxel and vinorelbine tartrate decrease the radiation/temozolomide-induced invadopodia activity of GBM cells [103]. Another study identified the ion channel drugs flunarizine dihydrochloride, econazole nitrate, and quinine hydrochloride dehydrate to reduce the radiation/temozolomide-induced invadopodia activity in GBM cell lines [107].

A phase II clinical trial on relapsed glioblastoma patients demonstrated that inhibition of CD95/CD95L signaling with APG101 in combination with RT shows efficacy [108]. Interestingly, in a glioma mouse model, APG101 prevented the formation of radiation-induced infiltrative tumor satellites and enhanced the efficacy of radiation therapy [86].

## 8. Conclusions

Most of the in vitro and in vivo studies presented in this review indicate that X-ray irradiation can trigger an increased migratory/invasive capacity in GBM cells. Within radiation oncology, there is still skepticism regarding the significance of these findings, mainly due to the lack of clinical data supporting the occurrence of these processes in GBM patients receiving RT. Tumor recurrence after RT can be explained by different causes such as intrinsic cellular radiosensitivity, hypoxic environment, and accelerated repopulation, among others. Currently, there is ample evidence to add altered GBM cell motility to this list [109]. An enhanced migratory and invasive behavior of GBM cells promoted by sublethal doses of irradiation may lead them to reach the border area of postoperative RT, escaping a cumulative lethal dose, forming the basis of locoregional relapse. Pharmacological strategies or new RT modalities that can diminish or inhibit the migration and invasion of GBM during RT are desired to improve the efficacy of therapy against this devastating disease [48].

## Figures and Tables

**Table 1:** In vitro studies investigating migration of GBM cell lines after low LET irradiation.

Cell Lines *	Dose	Migration Outcome	Type of Assay	Evaluation Time	Molecular Findings	Reference
U251	10 Gy	Increased	Scratch	24 h	Increased expression of MET oncogene after RT	[32]
U251, LN229	2, 4 Gy	Increased	Scratch	24 h	Upregulation of Notch signaling after RT	[33]
U251, LN229	2, 4, 8 Gy	Increased	Scratch	18 h	-	[34]
U251	8 Gy	Increased	Scratch	24 h	Enhanced cathepsin L expression after RT	[35]
M059K and M059J	2.46 Gy and 1.57 Gy ***	Increased for M059K, no effect for M059J	Scratch	24 h	Enhanced migration of DNA-PKcs-positive (M059K) but not DNA-PKcs-negative (M059J) cells after RT	[36]
SMA-560 (murine glioma)	2 Gy	No effect **	Scratch and transwell migration	8 and 24 h (wound healing), 16 h (transwell migration)	-	[37]
SNB19	2, 8 Gy	Decreasing tendency	Scratch assay and transwell migration	24 h	-	[38]
T89G, U87	2 Gy	Increased *	Transwell migration	Every 15 min	Activation of BK channels after RT	[39]
U87, LN229	2, 10 Gy	Increased *	Transwell migration	5 h	Increase in expression levels of ανβ3 and ανβ5 integrins after RT	[40]
U251	Conditioned medium of cells irradiated with 2 Gy and kept for 72 h	Increased	Transwell migration	22 h	Increased VEGF levels after RT	[41]
GL-15	35 Gy	Increased	Transwell migration	4 h	Increased expression of invasion-related genes (CXCL12, CXCR4, MMP2, MMP9, MMP12, EGFR, KCNN4, AP-1, ATF2, EGR3, REST) and KCA3.1 channels after RT	[42]
UN3, GM2 (primary human GBM)	Doses up to 8 Gy	Increased up to 6 Gy, decreased at 8 Gy	Transwell migration	48 h	Reduction in motility potential when inhibiting IGFR-1 signaling pathway upon RT exposure	[43]
U87	1, 3, 10 Gy	Increased (1 and 3 Gy), slightly decreased (10 Gy)	Transwell migration	24 h	Trend of increased expression of β3 and β1 integrin after RT	[44]
A172, LN229	5 Gy	Decreased	Transwell migration	18 h	MiR-10b enhances migration in presence of irradiation	[45]
U251, U373	2 Gy	Increased	Time-lapse videography	24 h	Increase in VEGF secretion after RTfor U373, no change for U251; synergistic effect between VEGF exposition and irradiation on migration velocity of U373	[46]
U-373 MG, U-87 MG	0.5, 2, 8 Gy	Increased for U-373 MG, no effect for U-87 MG	Time-lapse videography	24 h	Overexpression of Robo1 and Slit2 correlates with reduced motility Enhanced vimentin expression after RT	[47]
U87, U138, A172, LN229	6 Gy	No effect	Spheroid migration	Up to 50 h	Inhibition of β1 integrin diminished cell migration for both irradiated and non-irradiated cells	[30]
LN18, LN229, U87	3 Gy	Increased	Spheroid migration	Every 24 h for 4 days	Enhanced ανβ3 integrin expression after RT	[48]
U251, U87	8 Gy	Increasing tendency	Spheroid migration	24 h	Enhanced MMP-2 expression after RT; MMP-2 inhibition led to reduced migration	[49]
GaMg, U87	5, 10 Gy	Decreased	Spheroid migration	Every 24 h for 4 days	-	[50]
GaMg, U87	Ranging from 10 to 60 Gy (Fractionated)	Decreased	Spheroid migration	Every 24 h for 4 days	-	[51]

* Unless otherwise stated, the cell lines are established human GBM cells. ** No effect observed in vitro; however, in vivo an increased number of animals with satellite tumors was observed when tumors were irradiated when compared to a non-irradiated control group. *** Correspond to the iso-survival doses (ID50) of X-ray for M059K and M059J cell lines, respectively. h: hour.

**Table 2:** In vitro studies investigating invasion of GBM cell lines after low LET irradiation.

Cell Lines *	Dose	Invasion Outcome **	Evaluation Time	Molecular Findings	Reference
U251	10 Gy	Increased	24 h	Increased expression of MET oncogene after RT	[32]
U251, LN18	Conditioned medium of cells irradiated with 2 Gy and kept for 72 h	Increased	22 h	Increased VEGF levels after RT	[41]
U87, U251, and T98G	5 Gy	Increased	18–24 h	Inhibition of the invasive behavior after RT by a MDA-9/Systenin inhibitor	[52]
M059K and M059J	2.46 Gy and 1.57 Gy ***	Increased for M059K, no effect for M059J	24 h	Enhanced invasion of DNA-PKcs-positive (M059K) but not DNA-PKcs-negative (M059J) cells after RT	[36]
LN229, LN18 and U87	4 Gy	Increased for LN229 and U87, decreased for LN18	24 h	-	[53]
3 primary GBM cell lines and GL-15	35 Gy	Increased	24 h	Inhibition of the potassium channel KCa3.1 activity reduced the irradiation induced invasion	[42]
LN18, LN229, U87	1, 3, 6 Gy	Increased in a dose-dependent manner	24 h	Enhanced ανβ3 integrin expression after RT	[48]
UN3, GM2 (primary human GBM)	0–8 Gy	Increased until 6 Gy	48 h	Inhibition of EGFR, IGFR-1, PI-3K, and Rho signaling reduced the irradiation-induced invasion	[43]
U251, U87	8 Gy	Increasing tendency	24 h	Enhanced MMP-2 expression after RT; MMP-2 inhibition led to reduced invasion	[49]
U251	2 Gy	Increased	24 h	Increased MMP activity after RT	[54]
U87	3 Gy	Increased	20 h	Enhanced MMP-2, MMP-9, and VEGF expression after RT	[55]
U251, U373, LN18, LN428	1–5 Gy	Increased in cells lacking functional PTEN (U251 and U373) no effect in cells harboring WT-PTEN (LN18 and LN428)	24 h	Enhanced MMP-2 secretion after RT in cells lacking functional PTEN, but not in cells harboring WT-PTEN	[56]
U251	8 Gy	Increased	24 h	Enhanced cathepsin L expression after RT	[35]
7 primary human GBM cell lines	4 Gy	Increased in 3 cell lines, no effect on the others	24 h	-	[57]
U87, U138, A172, LN229	6 Gy	No effect (spheroids embedded into a collagen type I matrix)	Up to 50 h	Inhibitors of JNK, PI3K, and p38 MAPK, significantly impaired invasive capacity	[30]
A172, LN229, LN18, U138	6 Gy	Decreased for A172, no effect to the others	24 h	Increased β1- and β3-integrin cell surface expression and MMP-2 expression after RT; treatment with anti-β1, anti-β3, or MMP-2 inhibitor reduced the invasive potential	[58]
U87, A172, U373 and U251	Up to 3 Gy	Slightly decreased for U251 at 1Gy, no effect to the others (co-culture assay, glioma invasion into a background of normal human astrocytes)	96 h	Inhibition of MMP reduced U251 invasiveness	[31]
SNB19	2 Gy, 8 Gy	Decreased	24 h	-	[38]
A172, LN229	5 Gy	Decreased	18 h	MiR-10b enhances invasion in the presence of irradiation	[45]

* Unless otherwise stated, the cell lines are established human GBM cells. ** Unless otherwise stated, Matrigel-coated transwell invasion assays with an 8.0 μm porosity were used. *** Correspond to the iso-survival doses (ID50) of X-ray for M059K and M059J cell lines, respectively. h: hour.

**Table 3:** In vivo studies investigating the invasive behavior of GBM after low LET irradiation.

Animal	Cell Line	Irradiation Dose	Histological Findings	Molecular Findings	Reference
Rats	9L (rat glioma)	Cell pre-irradiation with 1 or 3 Gy	Increased invasive behavior for tumors growing from pre-irradiated cells	Increased MMP-2 and reduced TIMP-2 in tumors from pre-irradiated cells	[48]
Rats	9L (rat glioma)	Cell pre-irradiation with 1 or 3 Gy	Increased invasive behavior for tumors growing from pre-irradiated cells	Cell pre-treatment with inhibitors of IGFR-1, PI-3K, and Rho significantly reduced irradiation mediated invasion	[43]
Mice	LNT-229 (human GBM)	Single fraction of 8 Gy to a 7 × 7 mm field 21 days after tumor implantation	Formation of cell satellites distant from the bulk tumor when tumor is irradiated	Increased MMP-9 and HIF-1α observed in irradiated tumors	[78]
Rats	F98 (rat glioma)	A total of 15 Gy, whole brain, 14 h prior to tumor implantation	Increased infiltrate surface of tumors and lower survival for animals with pre-irradiated brains	Increased TGF-b1; IL-1b; MMP-2; PGE_2_; PGD_2_; COX-2 in pre-irradiated brains	[79]
Mice	ALTS1C1 (mouse astrocytoma)	Single dose of either 8 Gy or 15 Gy to a 1 cm field 13 days after tumor implantation	Reduced tumor growth rate, but increased tumor invasiveness	Increased SDF-1 and HIF-1 on satellites of irradiated tumors	[80]
Rats	Primary patient-derived GBM cells	Single dose of 50 Gy to a target volume with 33mm radius on day 84 after tumor implantation	Increased proliferation and migratory/invasive behavior for irradiated tumors	Increased Ki-67; MMP-2, CD-44 in irradiated tumors	[81]
Mice	U87 (human GBM) (Bioluminescent)	Single dose of 6 Gy or five fractions of 2 Gy starting on day 14 after tumor implantation	Increased invasive behavior and higher number of tumor satellites when tumors were irradiated	No changes in MMP-2 between irradiated and control	[82]
Mice	U87MG-Katuska (human GBM)	Five fractions of 2 Gy starting on day 7 after tumor implantation	Increased infiltrative behavior for irradiated tumors	Upregulation of SDF-1, activation of BK channels after RT	[83]
Mice	U1242-luc cell (human GBM)	Four fractions of 2.5 Gy starting on day 11 after tumor implantation	Similar infiltrative behavior for control and irradiated groups.	Inhibition of the infiltrative behavior after RT by combination with an MDA-9/Systenin inhibitor	[52]
Mice	G7 (patient derived GBM cells)	Three fractions of 2 Gy to a 10 × 10 mm field 10–11 weeks post-tumor implantation	Increased infiltrative behavior for irradiated tumors	Inhibition of the infiltrative behavior after RT by combination with a MRCK inhibitor	[84]
Mice	U87 (human GBM)	Cell pre-irradiation (2.17 Gy) or, pre-irradiation plus genistein treatment	Increased invasive behavior for tumors growing from pre-irradiated cells	Cell pre-treatment with genistein blocks the DNA-PKcs/Akt2/Rac1 pathway, reducing irradiation-mediated invasion	[36]
Mice	U251 (human GBM)	Three fractions of 5 Gy to the pre-implanted tumor	Increased infiltrative behavior and more satellites for irradiated tumors	Increased expression of phospho-STAT3 (Ser727) and phospho-STAT3 (Tyr705) in irradiated tumors	[85]
Mice	SMA-560 (mouse glioma)	Single fraction of 6 Gy on day 4 after tumor implantation	Radiation-induced tumor satellite formation	Fewer tumor satellites when animals are treated with an inhibitor of CD95 ligand	[86]
Rats	F98 (rat glioma)	A total of 65 Gy to the right hemisphere 3 months prior to tumor implantation	Increased proliferation and invasiveness for tumors growing in pre-irradiated brains Lower survival for animals with tumors growing in pre-irradiated brains	Higher expressions of CXCL12, VEGF-A, TGF-β1, and TNFα in irradiated brain tissue Higher expression of CXCR4, FGF-2, and ERK2 in tumor cells growing in pre-irradiated brains	[87]
Mice	SMA-560 (mouse glioma)	Five fractions of 4 Gy to the right hemisphere 7 days after tumor implantation	Increased number of animals with satellite tumors when tumors were irradiated as compared to control group	No changes in abundance of Iba1+ or CD68+ reactive microglia and CD3+, CD8+ cytotoxic or FoxP3+ regulatory T cells between irradiated and control	[37]
